# Motivations, Barriers, and the Potential of AI in Physical Activity Apps for US Older Adults: Interview Study

**DOI:** 10.2196/96114

**Published:** 2026-10-16

**Authors:** Abhinav Choudhry, Jonathan Mshvelidze, Sean P Mullen, Devorah Kletenik, Rachel F Adler

**Affiliations:** 1 School of Information Sciences, University of Illinois Urbana-Champaign, 501 E. Daniel St. MC-493, Champaign, IL, 61820, United States, 1 (217) 333-3280; 2 Macaulay Honors College, City University of New York, New York, NY, United States; 3 Department of Health and Kinesiology, College of Applied Health Sciences, University of Illinois Urbana-Champaign, Urbana, IL, United States; 4 Computer and Information Science, Brooklyn College, City University of New York, Brooklyn, NY, United States

**Keywords:** older adults, mobile health, mHealth, physical activity, generative AI, GenAI, wearables, human-computer interaction, technology acceptance, qualitative research, conversational agent

## Abstract

**Background:**

The older adult population is increasing rapidly and experiences a multitude of chronic conditions affecting quality of life. Physical activity and reduced sedentary behavior reduce adverse health conditions and help preserve functional independence and cognitive abilities, but the older adult age group has much lower levels of physical activity than recommended compared with other population demographics.

**Objective:**

The study aimed to understand older adults’ motivations toward health and physical activity and the role of technology in achieving their health and physical activity goals and to explore the attitudes toward the use of generative AI in health apps.

**Methods:**

This study was qualitative and based on hour-long interviews with 20 older adults living in the United States. To elicit specific design preferences, participants were presented with visual probes of AI-generated avatars. The interviews were analyzed using thematic analysis, and the coding process drew on reflexive and grounded theory techniques.

**Results:**

Older adults primarily wished to maintain functional independence, both physical and cognitive, prioritizing baseline maintenance rather than continuous optimization. They reported satisfaction with levels of physical activity, adjusting them to chronic conditions and injuries. They were active through everyday functions of living as well as structured exercise programs with many participants using wearable devices and smartphones to help monitor their activity. They reported being motivated by social accountability and gamification, although some found the latter condescending or anxiety-inducing. Accessibility issues were mentioned frequently, including problems with touchscreens, limited viewing, difficulty navigating interfaces with clutter, complex multitouch processes for undertaking actions, and steep learning curves. Computers were preferred for certain tasks over mobile phones due to easier viewability, keyboard access, and greater security features. Personalization was highly desired in health apps. There was a willingness to use more health technology, including generative AI, and they were willing to consider AI in both motivational and informational roles in apps as long as it was personalized. AI embodiment preferences varied widely but with an emphasis on relatability. However, there was marked skepticism toward data privacy, concerns about the loss of independence and self-efficacy, and doubts about AI’s accuracy.

**Conclusions:**

Older adults are primarily motivated by sustaining functional independence, although accessibility barriers in current health apps remain. Participants were open to AI for physical activity support, but expectations varied. They viewed AI as a passive “tool,” a directive “coach,” or a relational “companion.” Considerable variation in preferences for gamification and visual design suggested that future apps require greater modularity. As skepticism over data privacy and AI accuracy remains high, greater transparency and explainability might be needed to engender trust.

## Introduction

### Older Adults and Physical Activity

The demographic composition of the global population is trending toward a higher proportion of older adults, a shift that presents significant public health challenges across the world [1]. According to the 2020 Census, the US population aged >65 years stood at 55.8 million, or 16.8% of the general population [2]. This demographic grew nearly 5 times faster than the total population over the 100 years from 1920 to 2020 [2]. Older adult populations commonly experience a multitude of chronic conditions that accrue with age and also worsen due to decreasing physical activity (PA) with age [3]. PA and reduced sedentary behavior have proven benefits for older adults, including the prevention of chronic disease, maintenance of functional independence, and preservation of cognitive health [4]. Despite these known benefits, a large portion of the older adult population in the United States does not meet recommended PA guidelines [5], making the development of effective, scalable interventions for sustained PA in older adults increasingly important.

### Technological Changes in Digital Health

Recent rapid developments in technologies have resulted in increasing applications to the health domain. Digital health is an umbrella term for all uses of digital technologies to improve health and well-being, and it includes computer-based, remote, mobile, and ubiquitous solutions [6]. On the whole, digital interventions have been found to be effective in PA interventions for older adults across many studies in the past few years [7], primarily by facilitating continuous self-monitoring and reducing logistical barriers to exercise. Such technologies, including smartphone apps, wearables, and AI-powered conversational agents, have shown the potential to be scalable and cost-effective solutions [8]. Real-life applications have also been employing gamification: the use of game design elements in nongame contexts [9]. Gamification has been found to be beneficial in the health domain [10] and shown to help older adults in having higher levels of motivation, enjoyment, and engagement while performing PA [11].

### Research Gap Motivating This Study

However, a persistent gap has been observed between the high potential of these tools and their low adoption and sustained engagement by older adults [12]. This suggests a fundamental disconnect between the technological solutions offered and the actual needs, motivations, and practices of this user group. A recent systematic review found that limitations in capability, motivation, and social support were principal in limiting the uptake of mobile health (mHealth) solutions among older adults and suggested blended approaches of face-to-face interactions with technological interventions as a way to improve adoption [13]. Other research has identified 10 factors as determinants of older adults’ adoption of mHealth: value, usability, affordability, accessibility, technical support, social support, emotion, independence, experience, and confidence [14]. Tailoring is also associated with much greater adherence rates [15]. Current older adult fitness technologies may also be lacking alignment with the needs and preferences of older adults [16].

To better understand their needs and preferences, we conducted interviews with 20 older adults to answer the following research questions:

1. What are older adults’ foundational health goals and primary motivations regarding PA?
2. What are older adults’ current practices regarding existing health technologies (eg, smartwatches and fitness apps) and what specific accessibility barriers do they experience?
3. How do older adults perceive the hypothetical use of AI-powered conversational agents for PA, and what specific roles and trust-related concerns do they envision?

### Background

#### The mHealth Technology Landscape

The mHealth landscape for older adults extends beyond the relative ubiquity of smartphones and wearables to include tablets and emerging unobtrusive monitoring technologies. While personal mobile devices are effective for short-term PA promotion, their long-term utility is often limited by high abandonment rates once the novelty fades. There is also a persistent digital divide in active usage. Although hardware ownership is rising, particularly among highly educated older adults, sustained engagement remains a challenge across the demographic [17,18]. To mitigate the visual and dexterity deficits associated with these smaller screens, research demonstrates that tablet-based interventions can offer distinct usability advantages, allowing older adults to engage more effectively with complex health dashboards due to the larger screen real estate [19]. Furthermore, the landscape is shifting toward ambient computing and voice-first interfaces, which offer a lower barrier to entry for users with limited mobility or technical literacy [20].

#### Accessibility and the Limits of Usability

Historically, low adoption of mHealth technologies among older adults was attributed to physical and cognitive barriers. Systematic reviews confirm that age-related declines—specifically in manual dexterity, visual acuity, and cognitive processing speed—create significant hurdles for standard smartphone interfaces [12]. Wildenbos et al [21] demonstrated that basic interface deficits, such as small touch targets, complex navigation hierarchies, and insufficient contrast, can render an app functionally unusable regardless of the app’s intent. However, addressing usability alone does not guarantee engagement. Foundational information systems theory, specifically the senior technology acceptance model (STAM), confirms that for older adults, attitude and utility ultimately mediate the impact of physical limitations [22].

#### A Look at Engagement: Foundational Techniques and Gamification Strategies

To drive engagement beyond simple utility, gamification is frequently used to foster motivation. While substantial evidence supports its general efficacy in improving health outcomes [23], the evidence specifically for older adults is less robust [10]. Considering self-determination theory (SDT), mechanics designed to drive extrinsic motivation (eg, points, badges, and leaderboards) may fail to sustain engagement if they distract from the direct insight provided by self-monitoring or fail to support the user’s intrinsic health goals. Tabak et al [24] illustrated this risk, noting that specific game elements can sometimes weaken the correlation between the app and the actual health goal if they distract from the direct insight provided by self-monitoring. Consequently, it is argued that gamification for this demographic must be applied with “adaptive engagement,” allowing users to customize motivational affordances to their specific needs [11].

#### Social Connectedness Paradox and Privacy Barriers

Social support is widely recognized as a determinant of PA, but digitizing it presents unique risks; purely cognitive behavior change techniques are often less suitable for older adults compared to meaningful social support [25]. This uncertainty is often driven by a virtual paradox of connectedness: while older adults desire connection, they frequently reject the specific digital mechanisms used to facilitate it. This tension can be understood through socioemotional selectivity theory (SST), which posits that as time horizons shrink, older adults prioritize emotionally meaningful relationships with close friends and family over knowledge building with peripheral acquaintances [26]. Consequently, mHealth features that broadcast data to a wide, impersonal network conflict with this preference for selective intimacy. Orji [27] found that while competition can foster commitment, it introduces significant risks for this demographic, including heightened anxiety regarding self-esteem. Privacy barriers further compound this issue; older adults are significantly more likely to raise concerns about data confidentiality [28]. As a result, forced digital interaction can alienate users, who often prefer offline socialization, such as discussing results privately with family [24], over built-in cooperative elements that appeal only to specific, highly social user types [29].

#### Social Presence of Conversational AI

Conversational agents (CAs) are evolving rapidly, and those with unconstrained natural language input yield high user satisfaction and improved health outcomes, particularly in chronic disease management; they can foster a relationship similar to human coaching, where the natural language modality itself builds rapport [30]. This is supported by findings that the act of vocalizing health goals to an agent encourages psychological commitment [31,32]. Humans subconsciously apply social rules to computer interfaces, and this tendency toward anthropomorphism is particularly pronounced in older adults, who often use human social scripts to navigate digital interactions [33,34]. Embodiment appears to be helpful in gaining trust and acceptance among older adults [35,36]. An embodied virtual representation, commonly called an “*avatar*,” can be a tool for persons for mediating communication and to derive meaning from interactions with the environment [37]. This perception as social entities increases engagement but introduces design risks [38]. Hyperrealistic avatars can elicit “uncanny valley” discomfort, while cartoonish representations may be perceived as patronizing [39]. Concepts of homophily suggest a preference for peer-like avatars to facilitate trust [40], and recent research affirms that properly designed peer-like CAs are effective partners in increasing PA and dietary behaviors [41].

#### The Research Gap

The existing literature is extensive but fragmented, often examining domains such as usability, gamification, and trust in isolation. Furthermore, the prevalence of interventionist studies creates a bias toward evaluation, where user insights are constrained by the specific features of a prototype rather than revealing the user’s broader context. Consequently, there is a lack of comprehensive research that synthesizes the relationship between an older adult’s foundational health goals, their existing technology habits, and their trust requirements for future AI interactions. To bridge this gap between current behaviors and future expectations, this study adopts a multitheoretical lens. By integrating the STAM for usability, SDT for motivational goals, and SST for social and privacy preferences, we conducted interviews with older adults about their attitudes toward PA, current PA practices and barriers, and their perceptions of an AI-based tool for supporting PA.

## Methods

### Participants and Recruitment

To meet study inclusion criteria, participants had to be 65 years of age or older, reside in the United States, and speak conversational English. Recruitment was conducted through university email listservs, flyers, and the newsletter of a prominent lifelong learning institute for older adults. Interested participants completed a demographic questionnaire to determine eligibility, and the research team screened them based on responses. After every fresh outreach effort (listserv, flyer, or newsletter), interviews were scheduled with all eligible participants. Thereafter, a fresh outreach effort was made until the target sample size (n=20) was reached. A total of 30 unique respondents filled out the screening survey, but only 28 individuals were eligible, of whom 20 finally consented to being interviewed.

### Data Collection and Procedure

Interviews were conducted between May 2024 and January 2025 via videoconferencing software (Zoom; Zoom Video Communications) by the primary researcher. The sessions followed a semistructured format lasting approximately 1 hour. All interview data regarding PA and technology usage relied on participant self-report. The interview protocol was developed following guidelines for qualitative research questions [42] and covered health technology usage, challenges, and goals (Multimedia Appendix 1).

To elicit specific design preferences regarding AI embodiment, participants were presented with a series of visual probes. These consisted of AI-generated images (created using DALL-E models [OpenAI]; see Multimedia Appendix 2) depicting potential “*health coach*” avatars in fitness-oriented environments. The stimuli included the following:

- Realistic human avatars: young male, young female, older male, and older female.
- Robot avatars: a smiling bipedal robot and a plain bipedal robot.
- Animal avatars: a realistic dog, a cartoonish dog, a realistic cat, and a cartoonish cat.

These were intended to give participants a variety of choices in avatars. The clothing (sportswear) and background (sports field) of the avatars were selected so as to be associated with PA. There were no other selection criteria for the selected avatars, and the first image generated by the prompt was accepted as long as it followed the prompt accurately (eg, a prompt asking for a cartoonish dog got a cartoonish dog as a response and not a real dog image). Prompts used are provided in Multimedia Appendix 2.

Interviews were recorded on Zoom and transcribed using automated transcription services (Zoom and Whisper AI [OpenAI]), then checked for accuracy by the primary researcher.

### Data Analysis

Data analysis used a hybrid inductive approach. The overall framework was reflexive thematic analysis as described by Braun and Clarke [43], elected for its flexibility in identifying overarching patterns across a heterogeneous dataset. Within this constructivist framework, we adopted specific coding procedures from the grounded theory tradition [44]—namely open line-by-line coding—strictly as practical tools for systematically building initial codes from the data upward. Acknowledging the epistemological debates between these methods, this pragmatic combination [45] was chosen specifically because the structured coding of grounded theory facilitated deep data familiarization, which then transitioned fully into the interpretive, theme-building processes of reflexive thematic analysis [43,46], without committing to a positivist theory-building paradigm.

The analysis proceeded in 3 stages. First, transcripts were subjected to initial coding to identify initial concepts. Every recorded video was viewed with the transcript in hand during this stage. Second, iterative grouping was applied to organize these codes into broader categories and explore relationships between them. Finally, these categories were synthesized into the final themes reported. To ensure rigor, initial coding was conducted independently by 2 researchers across the entire dataset (n=20). The researchers then brought their independent coding together, and contrasting perspectives were explored through collaborative reflexive dialogue in regular meetings to develop richer, multifaceted conceptual themes. Throughout this process, researchers maintained detailed reflexive notes. The process was conducted and tracked by hand, with pen and paper, whiteboard, sticky notes, and Microsoft Excel to ensure dependability and confirmability.

Thematic sufficiency was assessed informally by monitoring whether new interviews introduced significantly novel concepts. By the final 5 interviews (P16-P20), no new codes were generated during initial coding, suggesting thematic sufficiency within this sample [47]. However, we acknowledge that the demographic homogeneity of our participants may have contributed to earlier convergence and that saturation might not hold if sampling were extended to more diverse populations.

To categorize how participants imagined AI supporting their health and PA, our thematic synthesis organically generated an inductive typology based on participants’ primary orientations toward the agent’s role. Data interpretation was actively shaped by the coding team’s diverse positionalities. The primary researcher had an eclectic multidisciplinary background across computer science engineering, finance, and other disciplines, coupled with personal experience as a caregiver for an older adult. Furthermore, an extensive experience of interviewing individuals in both industry and academic settings, personal engagement with structured exercise routines, nondigital heuristics, fitness apps, and various AI models provided a nuanced understanding of the tools being discussed. This combination had considerable impact on the semistructured interviews by shaping the conversation. The primary researcher’s understanding of older adults’ heuristics helped elicit deeper insights into technology usage and avoided viewing any struggles as skill deficiencies. Conversely, the secondary coder, a computer science major, brought a younger, digitally native perspective, while a practical background as a lifeguard provided a grounded lens on physical safety and fitness. During the “Four Eyes” evaluation, this contrast was highly productive. It established the credibility of our interpretations by preventing technical biases or overly sympathetic caregiving perspectives from overshadowing the older adults’ verbal accounts. Finally, our detailed demographic reporting allows readers to judge the transferability of these insights.

Building on this reflexive approach, our thematic analysis was intentionally theoretically sensitized by the multitheoretical lens introduced earlier. To maintain the inductive integrity of reflexive thematic analysis, initial codes were generated directly from the participants’ reported practice and perceptions. However, STAM, SDT, and SST provided the analytical vocabulary necessary to elevate these descriptive codes into our conceptual themes, ultimately shaping the resulting tool, coach, and companion typology.

### Ethical Considerations

This study protocol was approved by the University of Illinois Urbana-Champaign’s Institutional Review Board (project IRB24-0536). Informed consent was obtained from all participants prior to data collection. Qualitative data and interview transcripts were deidentified and assigned participant codes (eg, P1, P2) to ensure confidentiality. Participants received Amazon gift cards worth US $20 each as compensation for their time.

## Results

### Demographic Characteristics

Twenty participants (men: n=7, 35%; women: n=13, 65%) were interviewed regarding their experiences with health technology. The sample was highly educated, with all participants holding at least an undergraduate degree. The sample was relatively homogeneous in terms of ethnicity. All collected participant demographic data are presented in Table 1 and are self-reported.

**Table 1.** Participant demographics.

Participant code	Sex	Age group (years)	Education	Ethnicity
P1	F^a^	75‐84	Graduate degree	White
P2	F	65‐74	Graduate degree	White
P3	F	65‐74	Graduate degree	White
P4	M^b^	65‐74	Graduate degree	White
P5	F	65‐74	Postgraduate degree	White
P6	F	75‐84	Postgraduate degree	White
P7	F	65‐74	Graduate degree	White
P8	F	75‐84	Postgraduate degree	White
P9	M	65‐74	Bachelor’s degree	White
P10	F	≥85	Graduate degree	White
P11	F	65‐74	Graduate degree	White
P12	M	75‐84	Postgraduate degree	White
P13	M	75‐84	Postgraduate degree	White
P14	M	65‐74	Postgraduate degree	White
P15	F	65‐74	Graduate degree	White
P16	M	65‐74	Graduate degree	White
P17	F	65‐74	Graduate degree	White
P18	F	75‐84	Graduate degree	White
P19	M	75‐84	Postgraduate degree	White
P20	F	65‐74	Graduate degree	White

a F: female.

b M: male.

### Attitudes and Motivations Related to PA

#### Attitudes Toward PA

##### Self-Reported Activity and Satisfaction

A majority of participants (12/20, 60%) reported maintaining active lifestyles, although the structure and intensity of this self-reported activity varied widely. (Subjective satisfaction with one’s activity level may not necessarily correspond with meeting established PA guidelines.) Their PA regimens included structured exercises (eg, circuit training, resistance training, yoga, swimming, and sports) as well as lifestyle activities, such as gardening, walking the dog, and household maintenance.

An example of a highly structured, multifaceted routine was provided by P1:

*Right now, I work out with a trainer three mornings a week. I walk every day. I’m always on the go. So, I usually log about 10,000 steps a day*.

Others integrated activity into their necessary daily tasks; P6 said:

*I’m pretty active in the morning. I walk my dog twice... I do some gardening*.

Participants also reported satisfaction with their current level of PA. Their goal was maintenance, seeking to sustain their current baseline activity, rather than seeking continuous improvement. P8’s comment reflected a common sentiment:

*I’m not training for a marathon, but I get my steps in, I do my gardening... I feel good. At my age, I think I’m doing just fine*.

However, not everyone liked PA. P10 was happy with her low activity levels, listing multiple reasons for her dislike, including profound boredom (“It’s so repetitive. I’d rather read a book”) and sensory discomfort (“I just hate sweating”).

##### Pragmatic Adaptation to Physical Barriers

This satisfaction was maintained through active negotiation with the physical realities of chronic health conditions and past injuries. These ranged from bone and joint issues to heart issues and past surgery history. P2 described the impact of chronic issues affecting the type of PA:

*I’ve had high blood pressure since I was in my early 40 s...I wonder if I should be a little more gentle... I don’t want to have surgery on my neck.*

It also manifested in a continual process of adaptation involving finding new activities to replace those no longer safe to perform. P5 swapped hiking with water aerobics:

*I’d love to go on the long hikes I used to, but with the arthritis in my knee... I just can’t. So now, I do my water aerobics. You do what you can.*

### Motivations Aiding Health and PA

#### Functional Independence

Participants by and large were motivated with the aim of living a quality long life and preventing functional immobility. They framed having a certain level of functional fitness as a prerequisite to valued activities, such as being able to engage with family, avoiding emergency health circumstances, preventing cognitive decline, and also looking or feeling good. P13 framed it as a “straightforward” choice:

*Well, at my age you have to decide, do I want to live or do I want to die?*

PA was similarly linked to cognitive preservation; participants viewed exercise as essential for maintaining mental clarity alongside physical capabilities (especially P13 and P18). Rather than seeking continuous improvement, their long-term objectives focused on sustaining baseline health—such as retaining energy and speed (eg, P6) and avoiding hospitalization (eg, P5).

#### Familial Motivation

Participants expressed a strong desire to maintain their health to see their descendants grow (eg, P8). P20 vividly described how losing weight would allow her to safely engage with her family:

*I’ll be able to play with my grandson instead of getting down and feeling like I can’t get back up because it’s hard to get up when you have all this extra weight.*

#### Sense of Community

Most participants (12/20, 60%) cited social factors and motivations when discussing their PA. Participants liked working with other people such as their spouse, children, friends, or an exercise class.

The sense of community was crucial for some and helped with exercise adherence, providing motivation and even a sense of security. Participants noted that group settings, such as yoga or classes, instilled the discipline required to maintain routines (eg, P11), while also offering peer-monitoring that made them feel safe (eg, P20). P4 was very expressive in describing the value of community:

*The encouragement usually comes from enjoying the game with other people...[I] make sure I have enough good social activities for my health. I think that’s important for the mental side of health. Coming into contact with social groups and through activities that I do... and also with my children to socially interact with them*.

#### Competition

A healthy sense of competition helped some participants, but this was typically with close family members or even against oneself. P2 mentioned an example of competition between mother and daughter based on achieving the activity levels tracked by Apple Health, which are shown as rings.

*I found that I was more motivated when I was doing it with my daughter. She was sharing her rings, and I was sharing mine. I liked that competition*.

[P2]

While intimate competition within the family proved motivating, broad group-based competition was often rejected. Participants favored collaborative or self-referential tracking (eg, P9), actively disliking generalized leaderboards. As P11 noted regarding university-wide step challenges, comparing oneself against strangers “doesn’t motivate me, but against myself, yeah.”

One participant was motivated by optimizing physical health, focusing on proactively improving performance in competitive bodybuilding:

*We are not just messing around. We work out 6 days a week... It’s a lot when you’re competing.*

[P14]

#### Accountability and Routine

Having someone else to discuss one’s health objectives and progress with was listed as a key motivation by a number of participants, and this was often routine based. This accountability took various forms, ranging from formal weekly weigh-ins with nutritionists (eg, P3) to informal peer pressure from a spouse or “buddy” (eg, P14). Some participants even expressed a desire for “nagging reminders.”

For some, this accountability was maintained through rigid schedules rather than direct supervision, such as committing specific days of the week to racquetball or fencing (eg, P4). Others relied on self-styled, task-based routines as a proxy for tracking overall activity, measuring their physical output. For example, P7 measured it by daily gardening milestones:

*How long is the grass? How many weeds are in the garden? How many tomatoes and cucumbers have I picked today?*

Professional accountability from personal trainers or community instructors was highly valued for providing tailored motivation (eg, P17). More importantly, participants prized instructors who understood age-specific physiological issues and could proactively offer modifications for injuries or joint problems, ensuring the exercise remained both safe and effective (eg, P3).

### Health Technology Apps: Attitudes and Barriers

#### Attitudes

##### Willingness to Adopt

Almost half (9/20, 45%) of the participants were current users of health apps, while others had used them in the past. Of nonusers, several people felt optimistic about using them. For some, potentially life-saving features such as fall detection and continuous heart rate monitoring were the key reason for use, particularly for those living alone (eg, P1). Some participants were more likely to use apps integrated with their current ecosystem, such as Apple’s, essentially for the ease of compatibility with existing hardware.

Health apps were widely considered convenient for routine tracking. P6 preferred digital tools that automatically organized their data over manual methods such as writing in notebooks, noting that digital apps provided greater accountability (eg, P9). This convenience was further amplified by age-specific customization and seamless integration with existing services, such as health insurance platforms (eg, P3).

##### Unwillingness: Threat to Self-Efficacy and Feelings of Condescension

Six (30%) participants mentioned not wanting to track their health at all with reasons ranging from lack of need to it being effortful. A perceived loss of self-efficacy was a major barrier and participants actively resisted technology that felt overly prescriptive. P16 was emphatic:

*I want to feel that I’m in control of the phone, not the phone in control of me.*

It also felt condescending for P19:

*I don’t need a pat on the back. I know I’m doing it. I don’t need to write it down.*

App features were underused partly due to the feeling of loss of independence. P1 expressed this sentiment best:

*I don’t use a lot of the capacities... I don’t like that it tells me to breathe. I know to breathe... I was raised to be independent, so I don’t need some piece of software telling me that it’s time to do something unless I’ve set an alarm.*

### Barriers

#### Overview

All (20/20, 100%) our participants used technology regularly in their lives with many also possessing smartwatches (18/20, 90%) and voice-based personal assistants (8/20, 40%). Only 2 (10%) participants did not use smartphones, although they used computers. Nevertheless, many barriers and issues with technology were identified. These could be divided into accessibility issues and user preference mismatches. Although older adults aged ≥75 years reported more accessibility concerns, many aged 65 to 74 years also had similar concerns.

#### Accessibility: Physical Constraints

Accessibility issues proved to be a major barrier to usage. These ranged from physical constraints, such as arthritis affecting touchscreen use, to cognitive difficulties in learning new workflows. Small mobile interfaces actively hindered interaction, with participants struggling against illegible font sizes (eg, P18) and the physical pain of using tiny keyboards (eg, P9). Consequently, a significant number of participants explicitly preferred computers or tablets, seeking the physical affordances of larger screens and tactile keyboards (eg, P15). As P7 stated:

*I got myself a keyboard with larger keys...My fingers miss a lot, especially if I’m doing texting... [Computer’s more comfortable] because the print’s bigger... I can see more on the screen.*

While some participants preferred voice interactions to bypass typing difficulties (eg, P4), they often encountered recognition errors, noting that systems struggled to understand their enunciation and frequently transcribed commands incorrectly (eg, P2).

Furthermore, modern devices were often viewed as unforgiving. Heightened touchscreen sensitivity and shifting interaction modes led to hesitation in adopting newer hardware. P13 expressed his exasperation:

*Sometimes it’s annoying that the more features they put on the iPhone or any of these, the harder these buttons are to navigate. I’m always accidentally going somewhere where I don’t want to go... this is an iPhone 12. The reason I don’t have the 15 is my daughter has it and ... just do that and screens disappear and new stuff comes on. It’s too sensitive.*

[P13]

#### Accessibility: Cognitive Overload

Participants frequently indicated a strong preference for visual simplicity, frequently reporting feeling overwhelmed with complex menus (eg, P11) or excessive data visualizations such as multicolored line graphs (eg, P9).

Unfamiliar iconography compounded this frustration, sometimes leading to outright abandonment, as P1 recalled:

*When I go to an app that’s just filled with symbols and I have no idea what they are, I just turn it off and go to something else*.

Extraneous options and constant notifications further contributed to information overload, occasionally driving users to the brink of returning their devices (eg, P13). Participants viewed these interruptions as highly disruptive to their workflow:

*All these other things are coming up. Do you want to do this? Do you want to upgrade? Very distracting and very annoying... Let me just finish what I’m doing. Don’t overload me with too much. People that are older are not used to that level of overload*.

[P16]

#### Accessibility: Learning Difficulty and Complex Processes

Advancing age made learning more difficult for participants, leading to them expressing a marked preference for short and smooth learning curves (eg, P12). Multistep procedures were flagged as a specific barrier to adoption. P3 described this frustration:

*The only time I think it really gets difficult as if I can’t figure out what steps to take to get to what I want to do; if it’s too many steps... do this now do that now... there are 10 or 12 steps... The easier it is to access the information I want, the more likely I am to use the technology*.

[P3]

Convoluted navigation flows were censured, with some participants finding deeply nested tasks across multiple screens virtually impossible to complete. As P4 stated passionately:

*Navigation to me is one of the cruelest things if it’s not done correctly, especially for people that are a little older*.

#### Mismatch With User Needs

Apps were also criticized for not putting user needs first but instead prioritizing developer needs or lazily adapting study results:

*I just think it’s so distilled...not even the essence of what you need. It’s more like some coder, some developer, who might look at a study or something put down what they think should be in the app, not what you need from the app*.

[P5]

Participants strongly desired automated, minimal-effort setup processes for new wearables (eg, P4). When initial configuration proved too complex, users often had to surrender their independence and rely on younger family members for assistance before they could comfortably use the device (eg, P20).

### Health App Preferences

#### Gamification Preferences

The majority (14/20, 70%) of participants showed an affinity for gamification, considering it useful for motivation. Many features of gamification came up, ranging from levels and points to leaderboards and competitive streaks.

##### Motivated by Gamification

Tangible rewards, such as points tied to health insurance premiums (eg, P2) or daily step targets (eg, P17), provided clear milestones that drove users to finish their daily routines. Participants generally responded well to this positive, automated feedback (eg, P9).

The motivation provided by awards and the ability to share with family added a social dimension. Reference points for distance helped too. This was best expressed by P20:

*They would send those little award things. You’ve gone this many steps, walked this many miles...it’s funny. In the beginning, I would send it to my kids. I said, “Look what I did.” I was all excited, Oh, my gosh! I mean, it was kind of neat because it made you feel like you’ve gone this far already, and you don’t think of it when you’re doing it... It was nice. It’s silly things, but it was just kind of neat to see*.

[P20]

While some users thrived on the interpersonal competition of leaderboards (eg, P16) or the discipline of maintaining multiday streaks (eg, P17), others found deep motivation in personal, visual targets. The visual appeal of closing daily activity rings drove direct behavioral changes, as P19 confessed:

*I like the [Apple Watch] rings... I want to close my rings. So, I will walk around the kitchen island... to get my steps in to close my rings... It’s a bit silly, but it works*.

##### Unmotivated by Gamification

Conversely, 7 (35%) participants explicitly stated that they were unmotivated by gamification, dismissing such features as childish, distracting, or irrelevant to their intrinsic health goals. Its purported usefulness still failed to make them comfortable with it (eg, P12) and some even called it inherently unsuitable for their demographic, as expressed by P6:

*I don’t think people my age think in those terms [levels, badges, challenges].*

Excessive focus on gamification was also regarded as a source of anxiety. A quote by P8 illustrates this:

*One of the one of my goals for the last 10 years is not to be too judgmental. I have friends who are really disappointed when they don’t get their 10,000 steps... where it becomes worrisome to them... the worrying is worse than not getting the 10,000 steps in.*

### Privacy Preferences

A quarter (5/20, 25%) of participants expressed very strong concerns about use of their data and privacy, citing fears of hacking (eg, P4), the commercial exploitation of even ostensibly anonymized personal data (eg, P5), and the financial extortion targeting their age demographic (eg, P13). This often manifested as a visceral and pervasive feeling of unease because the technology was literally “listening in.” P13 was cogent:

*[Siri] gives me the feeling that I’m being watched... I don’t know who’s all in on that conversation. The same with Amazon*.

### Informational Preferences

When seeking health information, participants used a sophisticated, multisource approach characterized by conditional trust. This often involved a rigorous verification process for search engine results, where participants explicitly avoided relying on top hits alone (eg, P8). P20 elaborated that the priority was honesty, even if it resulted in complexity:

*I always keep reading further... I'll read two, three things past that—sometimes it'll be the same... and sometimes it won't… it’s got to be honest. Some things cannot be simplified. It’s going to take some time and energy.*

However, this digital searching was balanced by participants who relied on nondigital sources. Participants grounded their trust in established medical authority (eg, P2), curated traditional media such as books and magazines (eg, P11), and their own somatic intuition, prioritizing what their bodies told them over external data (eg, P14).

### Attitudes and Preferences Toward AI in Health

The majority of participants (14/20, 70%) demonstrated a pragmatic willingness to try an AI-powered coach. Even in the case of skepticism, outright rejection was rare and participants were curious and wished to give it a chance to prove its utility.

### Role of AI

The analysis of participant preferences regarding the AI’s role revealed distinct patterns in how users negotiate autonomy and support. Participants viewed AI in different ways based on desired power dynamics: a relatively passive “tool” (n=9, 45%), a directive “coach” (n=8, 40%), or a relational “companion” (n=3, 15%). Figure 1 maps this visual typology, with individual participant classifications and supporting evidence provided in Multimedia Appendix 3.

**Figure 1.**
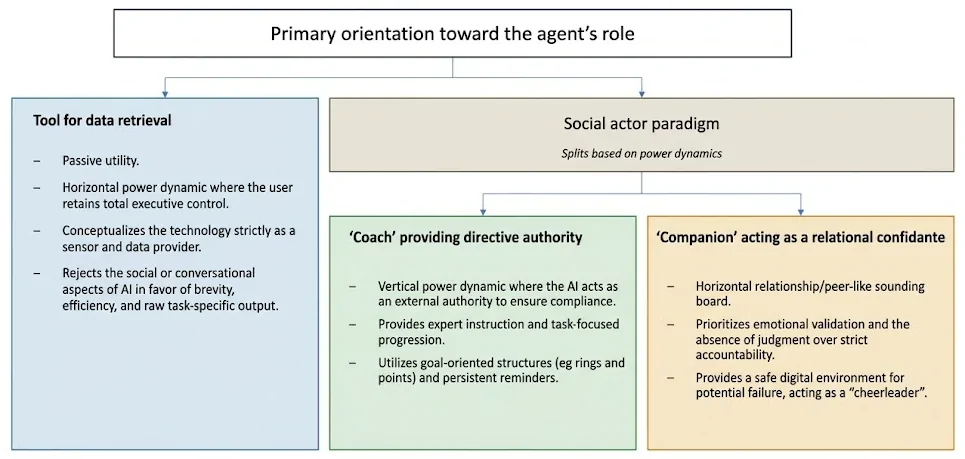
Visual typology of AI roles: mapping the tool, coach, and companion.

Many participants (6/20, 30%) were optimistic that an AI coach could provide essential accountability. A substantial number (n=6, 30%) explicitly desired that the AI coach “push” them, tracking specific metrics, such as workout frequency and weightlifting (as exemplified by P2). They preferred this push to be supportive rather than punitive, as P9 envisioned:

*If you had an AI app and there’s some avatar that you talked to who said “hey did you do your walk today? Well, why not?” I think encouragement rather than admonishment would be better*.

The access to exercise data on the move was considered helpful for influencing workout length and intensity. The use was informational but its hypothesized use in session also had the perspective of accountability. P3 felt it could be useful to track distances against targeted norms:

*If I was walking more than resting... am I going as far? ... Especially if I’m in a new place, like if I’m traveling and I don’t know the route as well.*

The facet of accountability was also present in pushing users to do more, as with a real personal trainer. P18 lucidly explained a future scenario of use:

*Especially if you’re wearing an Apple watch, it would know your heartbeat ... exactly how much exertion you’re putting into it. It could help keep track of your repetitions and give you advice... Let’s say, I said, I’m ready to quit, and if it said, Wait a minute. You need another set of repetitions on certain things that would be great, you know. I think it would help you do it would help make sure you got you got to, because it’s easy to say I’ve done enough when you don’t have a personal trainer there*.

[P18]

P11 was even more upfront about desiring an AI coach that was blunt rather than soft:

*As opposed to being nice and saying, “oh, I think you can do better.” No, I need someone that says, “come on, you’re half-assing it.” So, I don’t need a drill sergeant, but I need something more than a soft person*.

The personal monitoring could also be in the form of informational suggestions, such as for sleep and enjoyable activities (eg, P13). Participants valued the ability to ask follow-up questions in a “back and forth” dialogue with a CA. For some, this dynamic shifted the AI’s role from a strict instructor to a supportive peer. P8 explained:

*Coach is a little strong... I would like to think it is maybe a companion, an associate... somebody who is sympathetic, empathetic but holds me to it*.

On the other hand, some participants could only perceive AI as a utilitarian tool, viewing it as inherently impersonal. P3 felt that her interactions would be task-specific only:

*I think I would just be interested in the goal ‘tell me how far I’ve gone.*

She, however, appreciated the convenience of querying health statistics via natural language. Participants such as P10 wholly rejected the concept of an AI health coach for either motivation or information. Ultimately, even those receptive to AI motivation frequently alternated between viewing it as an active coach and a passive informational tool.

### Personalization

A strong desire for personalization was expressed by 11 (55%) participants. They wanted an app that would adapt to their specific health conditions and allow them to control their own goals. For instance, P1 wanted any advice to be catered to her situation, schedule, and current weather and not “generic walk 10,000 steps” advice. Similarly, P19 described desired flexibility in setting goals:

*I would want to be able to set my own goals... be able to go in and adjust it if I’m having a bad day*.

Others expressed interest in getting personalized notifications (eg, P3), while P13 and P17 were enthusiastic about guidance based on specific limitations, such as mobility issues. There was also a desire for a multistage CA that adapted its complexity to each user’s needs in “stages” proceeding gradually toward providing detailed research information (eg, P6).

However, some participants felt that current generative AI (GenAI) models lacked the contextual knowledge required to answer highly specific questions. P10 explained that desired specificity makes her prefer trusted websites over GenAI answers:

*All it could give me would be just generalisations. I’ve got specific questions. How much does it cost to do this? How many days would I have to wait for this? Google AI would have no way of knowing those answers*.

Some participants wanted very strong referencing of the information that the AI would provide. P6 noted that giving, “a detailed list of even academic references...would be perfect,” a sentiment echoed by P17 and P18. P5 wished that the AI be very “science based,” share citations carefully, avoid “dumbing down” information, providing very detailed reasons alongside a quick summary:

*I would want it to give me a reason...the important sources used to come to this conclusion... If you get that type of information, and they don’t put their citations with it, then you don’t know where the science is coming from.*

[P5]

When asked about preferences for a hypothetical assistant, most participants would prefer a conversational assistant integrated into a device they already use, such as a smartphone or smartwatch, rather than a separate, standalone device. A minority (n=2, 10%) explicitly preferred multiple devices, but the rest preferred it to be included within their phone. Regarding notifications, a small number (n=2, 10%) preferred a “pull” model (user-initiated), whereas 4 (20%) participants explicitly valued “push” reminders as a necessary mechanism for accountability.

### Visual Presentation

Embodiment was not a strong point of contention for most participants, with several explicitly expressing indifference toward it. However, among those who cared, 8 (40%) participants showed a clear preference for embodiment, while only 1 (5%) preferred a nonembodied CA. Furthermore, every participant showed at least some strong preferences among different avatar choices, displaying a clear preference for relatability. The majority (13/20, 65%) explicitly preferred human-like avatars, rejecting robot avatars for being unrelatable, using words such as “artificial” or “sci-fi” to describe them.

Within the human category, preferences were split by age. Some participants preferred older avatars, perceiving them as peers with shared experience while others preferred younger avatars, associating youth with up-to-date health knowledge, as in the case of P15:

*I would prefer the younger one... because they might be more up-to-date... and I might feel that they would be more encouraging*.

Beyond age, participants expressed a specific preference for physical attainability, often rejecting avatars with “sculpted” or hyperfit physiques as discouraging (eg, P18). P5 ridiculed the notion:

*I know zero ladies that have abs like that at her age*.

Similarly, P9 rejected aspirational avatars:

*If the app were some guy with a six pack... that’s just BS... I think more of a peer.*

This sentiment was not universal, though; in contrast, P17 wished to see aspirational physiques in the avatar:

*I’d like to see what they do if I can do that*.

The embodied version of the AI also had an impact on personalization. Some participants wanted to feel a connection with their AI coach. P13 expressed:

*I would like for it to more like a confidante... [Pointing to older male coach avatar] This picture feels like they’re interested in what I have to say. He’s the sort of guy I feel like I could talk with. I could sit down and have a beer with*.

### Skepticism and Distrust

AI evoked skepticism among many due to a number of reasons, the most important among them being accuracy and the “black box” nature of the technology. Threat to privacy and perceived loss of independence were other concerns.

Adverse previous experiences often led to concerns about poor accuracy. An illustrative experience was that of P4, who had played around with ChatGPT:

*The struggle I had when I was trying to get it to do exactly what I wanted it to do. A lot of times when it would fail at doing something, I didn’t know its limitations or it couldn’t figure out what I was trying to say*.

Similarly, P16 was “wary” for reasons of accuracy and information sources:

*It pulls up a lot of things from a lot of places. I don’t know how accurate everything is.*

P8 stressed the importance of accuracy over response speed:

*If you want to sacrifice detail, accuracy and completeness for efficiency—for making it quick and easy—you’re going to end up with a lot of stupid people, which we already have.*

The gradual creep of AI into daily life also caused discomfort, with some viewing it as an encroachment on independence. This discomfiture was expressed powerfully by P8 who questioned AI creators’ underlying motives:

*I want to question who’s putting this together, who is creating. Am I looking at Zuckerberg... Musk? Are these people that I would trust on other things? ... do they have a motive other than just information for me... I know Big Brother’s out there. I don’t the idea of having a device that would listen to everything I say or do bothers me*.

[P8]

Overall, there was a prominent lack of trust for AI among participants. Even those who had used a GenAI-based CA directly exhibited visible unease (eg, P20). Despite acknowledging its potential usefulness, P15 regarded herself as “suspicious” of GenAI and untrusting of its answers:

*When I ask a question I always go down further, because I don’t want to read the AI answer. I want to read the other answer*.

This caution frequently extended to fears regarding the prevalence of digital scams and phishing (eg, P9).

## Discussion

### Areas of Improvement in App Design for Older Adults

#### Matching Accessibility and User Needs

In this study, we found most participants to be current users of smart devices, but despite their comfort, there was a discrepancy between their general familiarity with apps and their actual comfort in practice, primarily due to accessibility issues. STAM [22] explains this discrepancy. When the procedural demands and learning curve of an app exceed users’ cognitive or sensory thresholds, the perceived utility of the technology collapses. This resonates with previous work done by Vaportzis et al [48] on older adults working with tablet computers. Even older adults who are adept current users of technology would prefer simplicity: their usage alone does not mean that the apps meet accessibility needs.

Participants in our study appeared to be developing adaptive strategies to cope with a fast-changing technological landscape in conjunction with their own cognitive and sensory declines. STAM explains this retreat to familiar desktop computers for substantive tasks as a rational calculation (not a digital deficit) with users actively filtering out complex apps to manage age-related barriers [22]. Excess features contributed to cognitive overload, creating a steep learning curve [48] that acted as a hard filter between basic device usage and mastering new health apps. This burden is compounded by hardware seen as failing to accommodate aging users [12]. While previous literature suggests a potential benefit for voice interfaces [32], our participants expressed mixed preferences, suggesting that giving users more interface options might be best.

Our typology also revealed that for some participants oriented toward the tool archetype, the demand for interface simplicity did not equate to a desire for simplified content. These expert users expected granular, high-level data, demonstrating that accessibility in mHealth should streamline navigation without diluting the scientific complexity of the information provided. This aligns with previous literature cautioning against oversimplification [14].

#### Social Connectedness

Among our predominantly well-educated sample, socialization and accountability were significant drivers, although whether this generalizes to more diverse populations warrants further investigation. SDT’s tenet of relatedness [49] illustrates why, for many, the exercise itself was secondary to the community interaction it provided. Facilitating these meaningful social connections acts as a vital relatedness-supportive mechanism [50]. Solitary app mechanics of most individual-centric mHealth tools fail to replicate this sense of communal security.

While digitizing social connection offers potential, it often conflicted with preferences for privacy and meaningful interaction. SST [26] accounts for this resistance, positing that as older adults prioritize emotionally meaningful relationships, mechanisms that broadcast personal data to peripheral networks conflict with their preference for selective intimacy. This theoretically contextualizes findings on social comparison by Koivisto and Malik [10] and Orji [27]. Thus, successful interventions would do well to shift from individual-centric models to community-centric ones that facilitate this safety net without enforcing high-pressure, privacy-invading competition.

#### Functional Independence

The older adults’ emphasis on maintaining a high degree of functional independence for their full lifespan also resulted in more pragmatic reasons for adoption of health apps or wearables. However, monitoring of step count and heart rate is known to have a contributing effect on increasing levels of PA [51]. It thus might be advisable to consider in mainstream health apps features for monitoring current chronic conditions to attract older users. Moreover, daily routines too warrant inclusion. Many participants got PA done as part of daily activities, such as walking their dog. An app would connect more with people if it discussed PA as part of their daily life rather than as workout routines alone.

Gamification contributed to encouragement for many participants and also added to accountability. This is encouraging given the proven effectiveness of gamification in improving health interventions for older adults [52]. However, extrinsic feedback loops in the form of tracking and gamification were also disliked by some participants despite diligence in performing PA. Here, SDT is explanatory because it predicts that those exercising higher autonomy will reject these as patronizing or irrelevant [49,53]. The taxonomy of motivational behaviors by Ahmadi et al [50] categorizes this exact friction as an autonomy-thwarting mechanism. Conversely, participants adopting the “coach” orientation actively requested strict accountability and persistent prompts. This is also consistent with SDT because actively choosing a directive system to enforce personal discipline remains an autonomous decision and is a delegation. The divergence of participant needs on the complexity of PA tracking measures—optimization vs simplicity—also has implications. PA apps aimed at the older demographic would thus do well to have a simple mode with limited tracking and reduced clutter while allowing for an expanded mode that affords detailed tracking, because complex apps have shown no additional benefit over simple pedometers for PA, as found in a systematic review and meta-analysis [51].

#### Personalized Interactions

The findings indicate that participants possess distinct and often contradictory requirements that necessitate personalization. The data reveal divergent design preferences that cannot be adequately addressed by a single, static app. A similar divergence appears in gamification preferences, where participants were quite polarized, although slightly more expressed motivation from game elements. Among those motivated by gamification, the underlying drivers varied significantly between those seeking competition and those interacting with features pragmatically to track habits. Those who actively rejected gamification viewed it as patronizing or irrelevant. A standardized strategy, such as a default leaderboard, thus risks actively demotivating the intrinsic and routine-driven groups. Avatar choices too are very divergent, and a wide range of choices are better if embodiment is used because individual preferences are strong.

These conflicting preferences suggest that adoption for this demographic is conditional on the ability to tailor the experience. Under SDT [49], the widespread desire to set one’s own goals reflects a fundamental need to preserve autonomy, driving participants to reject generic, prescriptive advice. Providing these meaningful choices operates as a core autonomy-supportive mechanism [50]. Consequently, implementing principles of autonomy support is critical, specifically the provision of choice and the minimization of external pressure [49]. Our findings also indicate that rigid system-defined metrics can further anxiety. Inflexible mandatory targets such as daily step counts can act as competency-thwarting features [50] that undermine user confidence. Therefore, streaks and targets that flexibly adjust to user circumstances may help avoid imposing undue pressure.

#### Future Implications for AI Integration and the Role of Trust

The study has a number of findings about the perception of AI that would be instrumental to future designers of apps using AI for aiding older adults in the context of PA. We found that participants were willing to try AI-based systems but nursed inherent skepticism. There are 2 major points of discussion concerning the use of AI by older adults. The first is on their hypothesized use cases, and the second is on the prerequisites that massively impact their adoption and perception of AI.

#### AI in Health Apps: Tool, Coach, and Sometimes Companion

The inductive typology that emerged from the data clearly aligns with functional distinctions in existing human-computer interaction literature. Specifically, it identifies whether participants viewed the AI as a “tool” for data retrieval [54], or following the social actor paradigm [33], as a “coach” providing directive authority [55] or a “companion” acting as a relational confidante [56].

The study shows that older adults are open to increased AI functionality in their apps. A remarkable proportion of the participants wished to feel a sense of being accountable to someone for their PA; even motivation could be attributed to discussions with an intelligent CA. Many use cases were informational but their presumed use was different from search engine behavior because they expected the CA to remember detailed personal context and provide information; this use case, although informational, is closer to that of a personal assistant for health. Previous literature too has found social support to be very helpful in increasing PA among older adults [57]. Their liking for gamification within apps was part of such motivation. However, gamification alone lacks a social component. While the need for social accountability is ideally satisfied by friends and family or even through personal trainers, many older adults lack them; it can be hypothesized that social support CAs could assist in such contexts, which could also help in alleviating loneliness [58]. Research has found that quality goal-setting practices, self-efficacy, and social support predict PA among older adults [59]. These can be facilitated by CAs, and this study too finds older adults to be willing to use them for these purposes. The CA having relatable embodiment and personality might help further.

However, based on our findings, health apps, especially with AI, have to take care not to negatively affect feelings of self-efficacy among older adults. We resonate with Klasnja et al [60] in that the AI should have the provision of modularity features—allowing users to use it as coach, tool, or companion. Providing this modularity ensures the app can dynamically satisfy the differing theoretical drivers of our participants—whether seeking accountability (SDT), relatedness (SST), or pure usability (STAM)—thereby protecting self-efficacy and respecting each user’s distinct needs.

#### Trust and Privacy as Foundational Prerequisites

Trust emerged as a foundational prerequisite for adoption, linking privacy concerns, AI skepticism, and information preferences. Under STAM [22], a lack of trust acts as a hard stop for adoption; if an app feels unsafe, its usability and intended utility are lost. Our participants viewed AI as a tool requiring human oversight rather than as an autonomous authority. This heightened sensitivity aligns with research indicating that older adults with higher educational attainment are significantly more likely to raise data privacy concerns than their peers [28]. The findings on information accuracy and privacy are corroborated by other studies [61].

Trust is also closely related to accuracy and algorithmic transparency. The participants’ distrust of AI stems directly from a practical awareness of GenAI’s propensity for hallucinations [62]. Inaccurate AI poses severe hazards in health care, and older adults require systems that make their reasoning visible. This demand aligns closely with explainable AI frameworks tailored for older demographics [63,64]. Participants expected the technology to justify its outputs rather than act as an unquestionable authority [64]. Their rejection of dumbed-down summaries serves as a practical test of this requirement. They consistently preferred referenced explanations over simplifications because obscuring the source data is often perceived as a breach of honesty. Designing these explanations to be both transparent and accessible [63] forms the foundation necessary to earn their trust.

#### Preliminary Design Considerations

On the basis of participants’ stated preferences and hypothetical reactions, we propose the following considerations for future designers of PA apps aimed at older adults. As these are derived from interview data and visual probe responses rather than observed usage behavior, they serve as preliminary guideposts rather than definitive rules.

1. Maintenance: Algorithms aimed at older adults may benefit from identifying a steady routine and rewarding consistency rather than aggressively pushing for continuous increases.
2. Social accounting: Designs could explore enabling easy sharing of physical activities and integrating social cues as a proxy for real-life social contact.
3. Relatability: More participants preferred age-appropriate avatars with average body types, suggesting these visual defaults may reduce psychological distance. This preference may be varied by cultural background and familiarity with digital agents.
4. Transparency: Providing direct links to reputable sources allows users to verify recommendations independently, supporting explainable AI principles.
5. Flexibility: Interfaces that support graceful failure states, such as allowing users to modify success criteria or auto-pause streaks during illness, better accommodate changing user physical realities.
6. Screen constraints: Developers might consider offering a companion view on tablets or desktops for complex data review while reserving mobile phones for passive tracking.
7. Passive data collection: Prioritizing automated data collection (eg, via wearables or sensors) can bypass the physical friction of manual entry.
8. Minimal interface changes: Participants expressed frustration with shifting layouts, suggesting that established navigation flows be changed only with substantial care.
9. Lifestyle integration: Rather than framing PA exclusively around structured workouts, apps should recognize and validate daily functional tasks (eg, gardening and housework).

### Limitations

This study has several limitations. The participant sample consisted predominantly of individuals in an urban area of the Midwestern United States who are well educated, relatively financially secure, and relatively ethnically homogeneous. Importantly, they possess a high baseline of technological literacy that likely exceeds that of the broader older adult population. As a result, key findings regarding how comfortable participants were with technology, their openness toward AI’s roles, and their specific skepticism toward AI may not apply to older adults with different educational, economic, or cultural backgrounds. We caution against overgeneralizing these findings; the nuanced views on AI observed here may represent a “best-case scenario” and cannot be automatically transferred to more diverse or underserved populations who face deeper digital divides. The study also relied on recall and self-reporting with participants reporting their own PA and technology use; an important caveat is that participants’ reported satisfaction with their PA levels may be influenced by social desirability, particularly given that they were recruited from an academic community and interviewed by university researchers. Self-report of PA is known to be susceptible to overestimation [65]. While participants expressed confidence in their activity levels, the absence of objective measures (eg, accelerometry) means we cannot confirm whether their activity meets recommended thresholds.

Finally, the discussion regarding AI agents was based on static visual probes and hypothetical scenarios rather than real-world use; participants’ trust and engagement might differ if they were using a functioning system over a long period. Visual probes in the future too could feature more diverse human avatars.

### Conclusions

In this study, we investigated the current usage of technology for PA among older adults and their attitudes toward AI usage. We found that older adults are primarily motivated by the desire to sustain functional independence and meaningful social connections. They sought maintenance of baseline health rather than chasing continuous improvement. While existing apps and wearables were widely adopted, rigid navigation flows, cognitive overload, and a mismatch with user needs continued to act as significant accessibility barriers. Most participants were willing to try AI-assisted technology but their expectations varied. They viewed it either as a passive user-led “tool,” a directive “coach” for strict accountability, or a relational “companion” for empathetic support. Older adults also possessed divergent views on gamification and tracking, and while the majority found it motivating, a significant minority found it condescending or anxiety inducing. Visual design preferences for CAs also varied widely, including split preferences on age, physique, and human versus nonhuman representation. Clearly, future AI apps should consider offering more modularity, personalization, and optionality.

Skepticism among older adults remained high due to pervasive concerns over data privacy, algorithmic hallucinations, and a perceived threat to user independence. AI that is personalized, transparent, and privacy protecting and that provides detailed referencing and explainable outputs when necessary is much more likely to be trusted. Greater use of AI-supported technologies for PA ultimately hinges on gaining user trust.

## Supplementary material

10.2196/96114 Multimedia Appendix 1 Semistructured interview protocol.

10.2196/96114 Multimedia Appendix 2 AI avatars and prompts used for avatar generation.

10.2196/96114 Multimedia Appendix 3 Typology of participant perceptions of AI in the physical activity context.

## Abbreviations

CA: conversational agent
GenAI: generative AI
mHealth: mobile health
PA: physical activity
SDT: self-determination theory
SST: socioemotional selectivity theory
STAM: senior technology acceptance model
