# Supplementary material for: Motivations, Barriers, and the Potential of AI in Physical Activity Apps for US Older Adults: Interview Study
Source: JMIR Aging. 2026 Oct 16;9:e96114. doi: 10.2196/96114 (PMC13674519; doi:10.2196/96114)
Supplement: Supplementary file 1 [file aging-v9-e96114-s001.docx]

# Multimedia Appendix 1

Semi-structured interview protocol.

Q1. What are some of your health goals?

Q2. How satisfied are you with the health goals you have set for yourself?

Q3. What do you think would be more useful for you: more information about health or more motivation?

Q4. Have you used any applications on your mobile or computer that are aimed at improving health? If so, could you tell us more about them. If not, is there any reason for not using them.

Q5. What are some health-related behaviours of yours that you are trying to change? What do you think would help you stick to your goals?

Q6. Could you tell us some previous experiences in which you had successes in including a health habit? What helped?

Q7. Could you tell us some previous experiences in which you were not successful in incorporating some health habit? Why do you think that happened?

Q8. If you ever used a health application, why did you use it and why did you stop using it?

Q9. Would you want to use a health application? What would you want the application to contain in terms of features?

Q10. What are some features that annoy you about technology and tech applications in particular, whether on computer or mobile?

Q11. What do you feel about fitness trackers? Have you used them? How has the experience been? If not, are you open to using them?

Q12. Do you play games, especially digital games? Could you describe your experience with games?

Q13. Have you interacted with personal voice assistants (Siri, Alexa)? Have you used conversational agents such as chatbots or ChatGPT?

Q14. Do you have hardware issues using phones, touchscreen, buttons etc.? Would you prefer having all functions on one device (a phone) or a separate physical device for different purposes?

Q15. Would you prefer a physical device for a conversational AI assistant/ helper/ mentor?
