# Supplementary material for: Motivations, Barriers, and the Potential of AI in Physical Activity Apps for US Older Adults: Interview Study
Source: JMIR Aging. 2026 Oct 16;9:e96114. doi: 10.2196/96114 (PMC13674519; doi:10.2196/96114)
Supplement: Supplementary file 2 [file aging-v9-e96114-s002.docx]

# Multimedia Appendix 2

AI Avatars and Prompts Used for Avatar Generation.

Please generate a full body image of a young female coach.

Please generate a full body image of a young male coach.

Please generate a full body image of a friendly robot coach.

Please make a smiling robot coach with full body visible oriented vertically.

Please generate a full body image of a dog coach.

Please generate a full body image of a dog coach which is more like a cartoon with full body visible oriented vertically.

Could you make an image of an older male coach who looks fit.

Could you make an image of an older female coach who looks fit and orient it vertically.

Could you make an image of a cat coach?

Could you make the cat coach look like a realistic cat?
