# Supplementary material for: Motivations, Barriers, and the Potential of AI in Physical Activity Apps for US Older Adults: Interview Study
Source: JMIR Aging. 2026 Oct 16;9:e96114. doi: 10.2196/96114 (PMC13674519; doi:10.2196/96114)
Supplement: Supplementary file 3 [file aging-v9-e96114-s003.docx]

# Multimedia Appendix 3

Table: Typology of Participant Perceptions of AI in the Physical Activity Context

| Participant | Demographics | Primary Orientation | Evidence | Classification Logic |
| --- | --- | --- | --- | --- |
| P1 | F / 75-84 / Graduate | Tool | *"I really don't want somebody, something telling me, now it's time to take a one-minute break... I know my body. So I personally don't need this stuff."* | Actively rejects AI as an authority figure or guide. She strictly wants passive utility (tracking steps, fall detection) and explicitly prefers a disembodied voice over a personified avatar. |
| P2 | F / 65-74 / Graduate | Coach | *"I would like to have, yes, someone who would say... How many times did you walk this week? How many times did you do stretching? How many times did you lift weights?"* | Desires a vertical power dynamic where the AI acts as an external authority to ensure compliance with specific tasks. She responds well to goal-oriented structures like rings and points. |
| P3 | F / 65-74 / Graduate | Tool | *"I think I would just be interested in the goal. Just tell me how far I've gone... I'm not a big chatter. So I wouldn't have lengthy, involved conversations with any sort of bot."* | Conceptualises the technology strictly as a sensor and data provider. Rejects the social or conversational aspects of AI in favour of brevity, efficiency, and raw task-specific output. |
| P4 | M / 65-74 / Graduate | Tool | *"I just prefer it to give me the information and me decide on what to do with it... I definitely wouldn't want it to say, 'you're not keeping up'."* | Desires an informational assistant similar to an objective database rather than a guide. Insists on a horizontal power dynamic where the agent provides data and the user retains total executive control over all health decisions. |
| P5 | F / 65-74 / Post-grad | Companion | *"You don't want to tell people when you've fallen short... I certainly wouldn't be ashamed to tell this app. It wouldn't judge me... and say, 'that's okay, try again tomorrow.' I need a cheerleader."* | Prioritises emotional validation and the absence of judgement over strict accountability. Seeks a safe digital environment for potential failure where the agent acts as a "cheerleader," providing gentle encouragement. |
| P6 | F / 65-74 / Post-graduate | Tool | *"It's more knowledge and less computer generated rewards, not interested... Like a professor or in fact like a doctor... Much more factual, historical, present day sense of how much activity you should be engaging in."* | Requires a high-level informational utility providing objective, historical data. Rejects behavioural interventions (points/badges) or social framing in favour of a professional, literature-backed interface focused on medical nuance. |
| P7 | F / 75-84 / Graduate | Coach | *"A friendly health coach... [I'd ask] how often. Repetitions. Sequential. So that you're improving, adding things to it each time to make it be progressive."* | Specifically seeks expert instruction and task-focused progression. Requires the agent to guide movement through modifications and sequential, step-by-step challenges rather than providing simple data monitoring. |
| P8 | F / 75-84 / Post-graduate | Companion | *"Coach is a little strong... I need a companion and associate... Somebody who is sympathetic, empathetic, that holds me to it... I don't want somebody looking perfect... I need a real person."* | Rejects vertical power dynamics and wants sympathy and empathy. Prioritises a horizontal relationship with a relatable, imperfect entity that acknowledges the difficulties of health maintenance without judgement. |
| P9 | M / 65-74 / Bachelor's | Coach | *"I think encouragement rather than admonishment would be better... a portion that's a little bit of a peer to sort of lubricate the conversation and then move into a coaching mode."* | Seeks an agent to solve self-described tendencies towards inertia through daily goal-tracking. Prefers interaction that is peer-like but also directive coaching at times. |
| P10 | F / 85+ / Graduate | Tool | *"I am immune to motivation. If I want to do it, I'll do it, but nobody's going to make me... I don't want a person talking about my health."* | Rejects AI in any social capacity. Ignores automated doctor texts and refuses to track steps. If forced to use an AI, she specifically selected a Robot avatar because she does not want a human-like entity judging or managing her health. |
| P11 | F / 65-74 / Graduate | Coach | *"I need an app that tells me that, that says, come on, you're half-assing it... I don't need a drill sergeant, but I need something more than a soft person."* | Desires a vertical power dynamic. Highly task-oriented and explicitly desires an AI that enforces strict compliance, pushes her past self-imposed mental limits, and demands rigorous accountability. |
| P12 | M / 75-84 / Post-grad | Tool | *"I don't feel I need a coach. I'm more self-motivated... I get in a habit pattern, and I try to keep it... You're talking about AI. That scares me."* | Completely rejects the Social Actor paradigm. Relies strictly on intrinsic motivation and physical paper logs, viewing advanced technology as a complex burden that disrupts his baseline quality of living. |
| P13 | M / 75-84 / Post-grad | Companion | *"I would like for it to have more like a... A confidant. He's the sort of guy I feel like I could talk with. I could sit down and have a beer with."* | Explicitly rejects the vertical power dynamic of a Coach, noting aversion to avatars that appear directive. Seeks a horizontal, peer-like sounding board that actively listens to him. |
| P14 | M / 65-74 / Post-grad | Tool | *"We're all just a science experiment of one... If you could develop a system that would have sensors and infer as maximal amount of information... and make it actionable insights."* | Relies on human coaches for actual guidance but uses tech (Oura rings, Withings scales) purely as a sophisticated array of sensors to track precise physiological data like HRV and REM sleep. Sees AI value as a data aggregator, fitting the Tool archetype. |
| P15 | F / 65-74 / Graduate | Coach | *"Someone encouraging. And but maybe a little bit pushy as well... some nagging reminders... I hit the 10,000 steps, it shows me a picture... I guess I go for them."* | Views the technology as an external manager responsible for keeping her on track. Utilises micro-accountability (prompting exactly 250 steps per hour) and explicit requests for persistent reminders. |
| P16 | M / 65-74 / Graduate | Coach | *"If it set a goal, if I reached the goal, that was some type of a reward... [I might ask] to compare to other people my age, what they're doing."* | While claiming to avoid competition, he reveals a reliance on external markers of success (Genius status in games). He seeks an AI that provides rewards and allows for normative comparison (benchmarking against peers). |
| P17 | F / 65-74 / Graduate | Tool | *"I'm all about the information. I would like it to say reference if I wanted to reference that medical journal... I would want the small short answers first. Then... the detailed."* | Views technology as a sophisticated sensor suite. Tracks granular metrics like VO2 Max and walking symmetry but explicitly rejects external nudges or alarms. She values factual, cited data over a personal relationship with the AI. |
| P18 | M / 75-84 / Post-graduate | Tool | *"If you added that function of a personal trainer... that would be great... I lean more towards the information, but it would be nice to have some motivational aspect of it, too."* | Primarily views AI as an analytical resource for technical exercise and diet guidance. Rejects the vertical power dynamic of human trainers who do not understand his limits, preferring a tool that provides raw data while he remains in executive control. |
| P19 | F / 75-84 / Post-graduate | Coach | *"I would want it to be... supportive and yet pushing, sort of like a personal trainer pushing you to do more... today I'd like to encourage you to increase your steps by 500."* | Explicitly desires a vertical relationship where the AI acts as a professional trainer. Recognises that her current self-guided activity lacks sufficient rigour and requests specific technical prods to hit target heart rates, expecting the AI for its ability to set higher standards. |
| P20 | F / 65-74 / Graduate | Coach | *"If I'd have to do it on my own I just don't do it... It's like when you're a kid in school and you get a gold star... I think like if there was something where somebody could be in touch with you after you miss."* | Relies on external guides (dietitians, teachers) for adherence. She explicitly requests the AI to initiate contact when she misses goals and expresses a strong affinity for gamified rewards like badges and stars. |
